# NRF2 and the Moirai: Life and Death Decisions on Cell Fates

**DOI:** 10.1089/ars.2022.0200

**Published:** 2023-03-16

**Authors:** Yoko Yagishita, Dionysios V. Chartoumpekis, Thomas W. Kensler, Nobunao Wakabayashi

**Affiliations:** ^1^ Translational Research Program, Fred Hutchinson Cancer Center, Seattle, Washington, USA.; ^2^ Service of Endocrinology, Diabetology and Metabolism, Lausanne University Hospital, Lausanne, Switzerland.

**Keywords:** NRF2, KEAP1, stem cells, cell differentiation, cell proliferation, programed cell death, cell signaling

## Abstract

**Significance:**

The transcription factor NRF2 (NF-E2-related factor 2) plays an important role as a master regulator of the cellular defense system by activating transcriptional programs of NRF2 target genes encoding multiple enzymes related to cellular redox balance and xenobiotic detoxication. Comprehensive transcriptional analyses continue to reveal an ever-broadening range of NRF2 target genes, demonstrating the sophistication and diversification of NRF2 biological signatures beyond its canonical cytoprotective roles.

**Recent Advances:**

Accumulating evidence indicates that NRF2 has a strong association with the regulation of cell fates by influencing key processes of cellular transitions in the three major phases of the life cycle of the cell (*i.e.*, cell birth, cell differentiation, and cell death). The molecular integration of NRF2 signaling into this regulatory program occurs through a wide range of NRF2 target genes encompassing canonical functions and those manipulating cell fate pathways.

**Critical Issues:**

A singular focus on NRF2 signaling for dissecting its actions limits in-depth understanding of its intersection with the molecular machinery of cell fate determinations. Compensatory responses of downstream pathways governed by NRF2 executed by a variety of transcription factors and multifactorial signaling crosstalk require further exploration.

**Future Directions:**

Further investigations using optimized *in vivo* models and active engagement of overarching approaches to probe the interplay of widespread pathways are needed to study the properties and capabilities of NRF2 signaling as a part of a large network within the cell fate regulatory domain. *Antioxid. Redox Signal.* 38, 684–708.

## Introduction

**FIG. 1.**
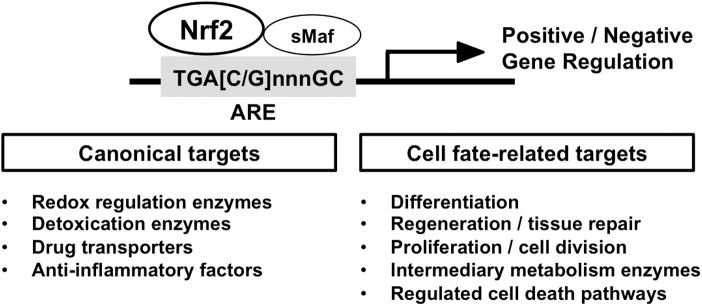
**NRF2 regulated genes affect multiple cell functions.** NRF2 is a transcription factor that translocates into the nucleus, heterodimerizes with sMAF proteins, and binds to ARE in upstream regulatory regions to activate or repress the transcription of a wide range of genes. Canonical NRF2 target genes encode enzymes related to redox regulation, xenobiotic and drug detoxication, drug transporters, intermediary metabolism, as well as anti-inflammatory factors. NRF2 target genes also include genes where direct transcriptional regulation by NRF2 leads to determination and/or fine-tuning of cell fates. ARE, antioxidant response element; Nrf2, NF-E2-related factor 2; sMaf, small Maf.

The transcription factor, NRF2 (NF-E2-related factor 2) belongs to the cap'n'collar (CNC)-basic leucine zipper (bZIP) family, assembling a complex with its heterodimeric partners, small MAF (sMAF) proteins, and binds to antioxidant response element(s) (ARE) to activate or repress a battery of NRF2-target genes (Fig. 1). In 1997, *Nrf2* null mice were first established, showing diminished expression of detoxifying enzymes, along with enhanced sensitivity to toxic xenobiotics (Chan et al, 2001; Itoh et al, 1997).

KEAP1 (kelch-like ECH-associated protein 1), a repressor facilitating the proteasomal degradation of NRF2, was described shortly thereafter as a critical factor affecting NRF2 fate and magnitude of cytoprotective responses (Itoh et al, 1999; Kobayashi et al, 2004). Based on these fundamental findings, NRF2 research accelerated the field of molecular toxicology to define its role as a critical defensive player against electrophiles and reactive oxygen species (ROS) (Kensler et al, 2007). The key components underlying the robust advancement of NFR2 research were (1) the generation and widespread dissemination of sophisticated genetic tools for modulating the structure and function of NRF2 and KEAP1 (and other accessory proteins) for studies *in vitro* and *in vivo*, and (2) the application of systematic transcriptomic and genomic sequencing methodologies.

Especially, comprehensive and high-throughput transcriptome profiling have continued to reveal an ever-broadening range of NRF2-target genes, including the many cytoprotective genes categorized as “canonical” NRF2 target genes, and some more recently characterized NRF2 target genes, such as cell proliferation-/differentiation-related genes and genes contributing to cell death pathways (Fig. 1). By now, over 300 potential direct NRF2-target genes have been reported, indicating the complexity of the biological functions of NRF2 in multiple biological settings (Malhotra et al, 2010; Yamamoto et al, 2018).

In this review article, we place a particular focus on the functions of NRF2 that affect cell fate determinations. Importantly, NRF2 target genes are tightly associated with the regulation of cell fate. In this regard, contributions to cell fate commitment are one of the key biological signatures of NRF2, which requires comprehensive and progressive understanding as we enter the new era of NRF2 research. We likened the cell life span to the human life course, which reminded us of the three goddesses (Moirai) in Greek mythology, who inescapably controlled the thread of life of individuals.

**FIG. 2.**
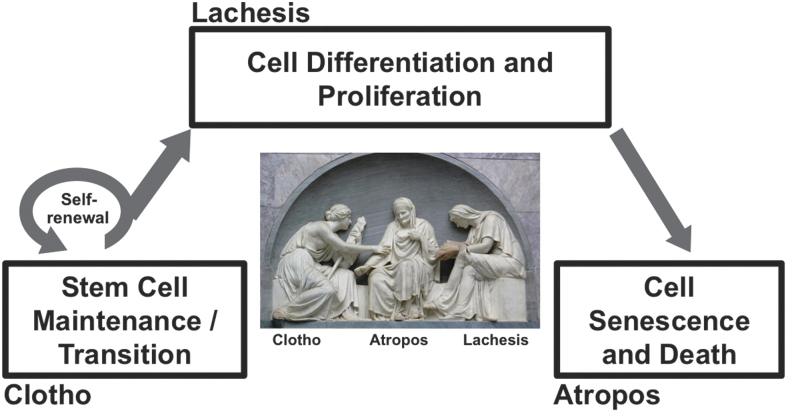
**Three Fates in the life cycle of a cell: Clotho, Lachesis, Atropos.** The major elements of the life cycle of a cell: (1) cell birth (stem cells), (2) cell differentiation and survival, and (3) cell death, are precisely regulated to direct the fate of individual cells. These phases can be compared with the Three Fates—the three Moirai (m-EE-r-eh) in Greek mythology who determine human destiny: the youthful *Clotho* spins the thread of life, the aged *Atropos* cuts it off—despite Clotho's attempts to prevent this—and *Lachesis* reads the allotment of each individual in the Book of Destiny. (Photo description: Grabmal des Prinzen Alexander von der Mark; Alte Nationalgalerie Berlin. Photo by Andreas Praefcke, Public Domain, https://commons.wikimedia.org/w/index.php?curid=2122291).

Given this inspiration, three phases of the cell life cycle, that is, cell birth, cell differentiation, and cell death, are compared with the Three Fates: *Clotho*, *Lachesis*, and *Atropos* (Fig. 2), where we summarize interactions between NRF2 signaling and regulatory mechanisms of cell fate determinations throughout their life cycle.

## CLOTHO: Cell Birth (Stem Cells)

### Embryonic stem cells (pluripotent stem cells)

Embryonic stem cells (ESCs) are found in the inner cell mass of a blastocyst after ∼5 days of development. They are pluripotent cells with indefinite self-renewal abilities and differentiation properties contributing to the three primary germ layers: ectoderm, endoderm, and mesoderm. In embryogenesis, the fertilized egg harbors totipotency, which is maintained in the zygote up to the eight-cell stage of the morula. Cell differentiation leads to a blastocyst composed of outer trophoblast cells and an undifferentiated inner cell mass. Although the inner cell mass loses totipotency, it retains the ability to develop into all cell types of the embryo, which is referred to as pluripotency. These cells are defined as ESCs (Wobus and Boheler, 2005).

The establishment of ESC lines derived from mouse embryos in the early 1980s, and that of human embryos a decade later are fundamental breakthroughs in the field of developmental biology. The role of NRF2 in ESCs has been investigated mainly *in vitro* using human and mouse ESC lines. The nuclear accumulation of NRF2 has been observed from the two-cell stage up to the blastocyst stage in the culture of harvested mouse zygotes (Lin et al, 2018). NRF2 expression is high in human embryonic stem cells (hESCs) and dramatically decreases on differentiation (Jang et al, 2014). These descriptive observations suggest a role of NRF2 in the earliest stages of embryogenesis and ESC fate determination.

Cell culture conditions (*e.g.*, passage number, oxygen tension) may confound interpretations of redox mechanisms in ESC fate. Lower-oxygen concentrations (∼1%–5%) than typical cell culture conditions (20% oxygen) have been reported to maintain ESC pluripotency, and reduce their differentiation in mouse and hESCs (Barbosa et al, 2012; Ezashi et al, 2005; Westfall et al, 2008). Excessive intracellular ROS and higher oxygen tension can be a trigger for ESCs to exit into a differentiation state (Yanes et al, 2010).

There is a fundamental importance of the physiological levels of ROS in cellular processes throughout the cell life cycle. The contribution of NRF2 signaling, a master regulator of the redox system, is beyond question. However, particularly in ESCs, the role of NRF2-mediated redox regulatory mechanisms in cell fate determination and cellular process has been investigated in only a few studies. Future studies should employ optimization of ROS stimulation and *in vivo* animal models, possibly through genetic engineering.

With these concerns in mind, the neurogenic effect of ROS in stem cells was examined using hESCs treated with paraquat. Elevated ROS levels resulted in acceleration of neuronal differentiation, which was enhanced by knockdown of *NRF2* (Hu et al, 2018). Another study demonstrated that mild oxidative stress provoked by exposure to glucose oxidase enhanced osteogenic differentiation and mineralization in mouse ESCs, which was inhibited by *Nrf2* knockdown (Sim et al, 2016). It was reported that silencing of *FTH1*, which encodes a major iron storage protein, activated NRF2 signaling and induced expression of metabolism-related NRF2 target genes, *G6PD* and *PGD* in hESCs (Scaramuzzino et al, 2021). Considering NRF2 as a transcription factor that modulates *Fth1* and also ferritin light chain (*Ftl*) transcription (Kwak et al, 2001; Thimmulappa et al, 2002), it could be assumed that the *FTH1*-NRF2 axis might contribute to iron homeostasis in hESCs, leading to metabolic changes.

**FIG. 3.**
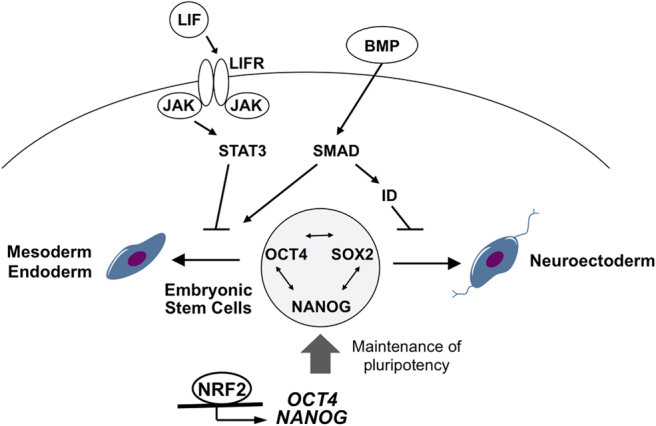
**Signaling networks in human embryonic stem cells.** Self-renewal and differentiation of embryonic stem cells are precisely regulated by complex signaling networks, which include the SMAD and JAK/STAT3 signaling pathways and pluripotency factors. NRF2 transcriptionally regulates core pluripotency factors, OCT4 and NANOG, in human pluripotent stem cells, thereby contributing to their maintenance. BMP, bone morphogenetic protein; ID, inhibitor of DNA-binding proteins; JAK, Janus kinase; LIF, leukemia inhibitory factor; LIFR, LIF-receptor; NANOG, Nanog homeobox protein; OCT4, POU class 5 homeobox 1; SMAD, suppressor of mothers against decapentaplegic; STAT3, signal transducer and activator of transcription 3.

Several signaling pathways and their underlying transcription factors are involved in the survival, maintenance, proliferation, and differentiation of ESCs as shown in Figure 3. NRF2 directly regulates proteasome activity at least partially through POMP, an essential molecular chaperon for proteasome assembly (Jang et al, 2014). NRF2-mediated high proteasome activity in hESCs seems to be important to regulate self-renewal and pluripotency of hESCs. A chromatin immunoprecipitation (ChIP)–seq study performed by Chorley et al (2012) suggested the potential transcriptional regulation of the *POMP* gene by NRF2.

Jang et al (2016) also demonstrated that NRF2 transcriptionally regulates the expression of core pluripotency-related genes. Under defined differentiation conditions, pluripotent hESCs are directed toward a mesendoderm or neuroectoderm fate, which is the first ultimate choice between the two cell fate commitments. Importantly, they showed that NRF2 binds directly to upstream regions of *OCT4* and *NANOG* to promote their expression. Together with other pluripotency-related factors, such as SOX2, the expression level of *OCT4* and *NANOG* regulated by NRF2 is suggested to suppress early cell fate commitment of pluripotent hESCs.

Si et al (2019) studied the effect of hyperglycemia on self-renewal ability of neural progenitor cells during embryonic development using a gestational diabetes mouse model. They showed that ciliary neurotrophic factor (CNTF), a key neuropoietic cytokine, regulates the imbalance between neurogenesis and gliogenesis caused by hyperglycemia. Moreover, it was shown that CNTF and NRF2 coordinately regulate neural development through pSTAT3, suggesting a potential role of signaling crosstalk between NRF2 and other canonical factors in the homeostasis of ESCs.

The signaling pathways, such as Janus kinase (JAK) pathway and bone morphogenetic protein (BMP), which have been reported to have direct- or indirect-crosstalk with NRF2 signaling in other cell types (Gong et al, 2020; Jiang et al, 2015b; Turei et al, 2013), are important players in the signaling network of ESC fate determination (Fig. 3). Hence, it could be hypothesized that NRF2 signaling may affect multiple signaling networks during the process of ESC fate determinations.

### Stem cell reprogramming

In general, mammalian cells undergo development to become more committed to their specific lineages. However, the generation of induced pluripotent stem cells (iPSCs) from somatic cells is often described as “a rewinding of the developmental clock.” An association of the NRF2 signaling pathway with somatic reprogramming might be inferred, but it is solidly unproven. The metabolic shift from oxidative to glycolytic energy production is a key molecular event during iPSC reprogramming. Hawkins et al (2016) studied the real-time activity of transcription factors during iPSC reprograming using a lentiviral reporter system that revealed a peak in NRF2 activity at day 8 of reprogramming.

This timeline corresponded with elevated ROS generation during the early stages of iPSC reprogramming, leading to the metabolic switch from oxidative phosphorylation toward glycolytic energy production. This metabolic switch seems to be supported by NRF2-mediated activation of hypoxia-inducible factor (HIF)1α as inhibition of NRF2 by KEAP1 overexpression in human iPSCs compromised metabolic reprogramming and resulted in reduced efficiency of iPSC colony formation. Jang et al (2016) used eight well-characterized iPSC lines, including human and chimpanzee lines, and examined the correlation between NRF2 signaling activity and differentiation bias of iPSCs (*i.e.*, a mesendoderm fate or neuroectoderm fate). It was demonstrated that iPSCs with lower NRF2 activity were more neurogenic rather than being directed toward a mesendoderm fate.

### Multipotent stem cells

Multipotent stem cells (MSCs), also called adult tissue stem cells, have been discovered in small numbers in most adult tissues, such as bone marrow, intestinal crypts, or adipose tissue. Although MSCs have a more limited potential to differentiate into various cells of the body compared with pluripotent ESCs, MSCs stand as key cells for implementation of biological events such as tissue development, regeneration, and repair. Studies of both loss and gain of function of NRF2 signaling provide insights into roles of NRF2 in MSC.

**FIG. 4.**
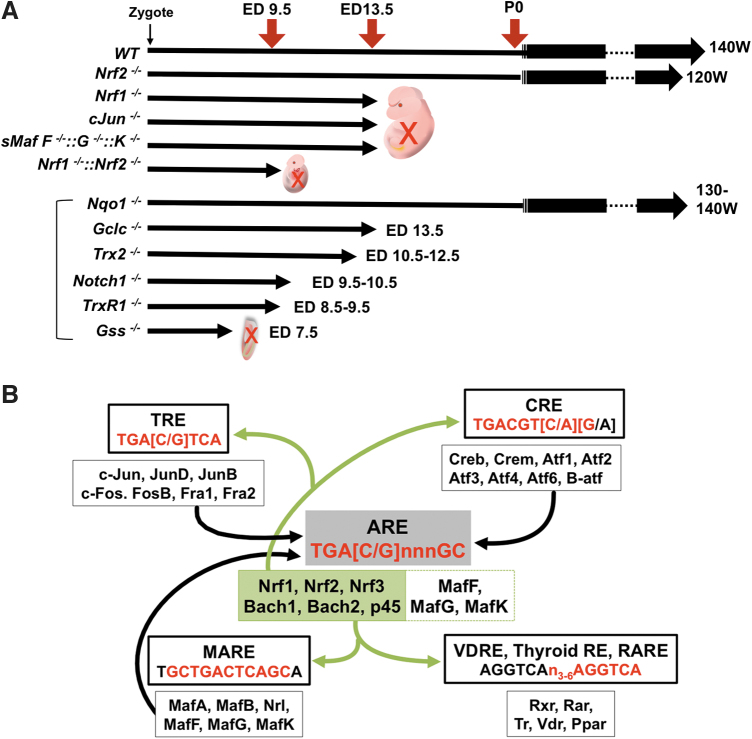
**Functional and molecular interplay between NRF2 and other CNC transcription factors in embryogenesis. (A)** During embryogenesis, diverse types of cells are generated from the fertilized egg, ultimately constituting each tissue and organ. The role of *cis*-element related transcription factors during embryogenesis has been investigated using loss-of-function strategies. Specifically, the observed lethal phenotypes highlight the biological significance of these transcription factors in embryonic development. The schema indicates the survival time of embryos for each loss-of-function model mouse of *cis*-element related transcription factors, as well as for selected NRF2-target genes. **(B)** The enhancer function of the *cis*-element “ARE” can be partially mimicked by TRE, CRE, and MARE elements and some nuclear receptor recognition elements. The lower boxes indicate the response element binding proteins, and the upper boxes indicate their corresponding binding sites. The core sequences in common with ARE and other factor recognition elements are indicated in *red*. ARE, antioxidant response element; ATF, activating transcription factors; BACH, BTB and CNC homology basic leucine zipper transcription factor; cJUN/JUN, Jun proto-oncogene; CNC, cap'n'collar; CRE, cAMP (cyclic adenosine monophosphate) response elements; CREB, cAMP-responsive element binding protein; CREM, cAMP response element modulator; ED, embryonic day; FOS, Fos proto-oncogene; FRA, Fos-related antigen; *Gclc*, glutamate-cysteine ligase, catalytic subunit; *Gss*, glutathione synthetase; MARE, Maf homodimer specific binding sites; *Nqo1*, NAD(P)H dehydrogenase, quinone 1; NRL, neural retina-specific leucine zipper protein; P, postnatal day; PPAR, peroxisome proliferator-activated receptor; Rar, retinoic acid receptor; RARE, retinoic acid response element; RXR, retinoid X receptor; Thyroid RE, thyroid hormone response element; TR, thyroid hormone receptor; TRE, TPA (12-O-tetradecanoylphorbol-13-acetate)-responsive element; *Trx2*, thioredoxin-2; *TrxR1*, thioredoxin reductase-1; VDR, vitamin D_3_ receptor; VDRE, vitamin D response element.

NRF2 signaling toward its canonical cytoprotective target genes is important to maintain integrity and survival of MSC. Accordingly, *Nrf2* null mice exhibit a shortened lifespan compared with littermate controls fed under *ad libitum* conditions in well-controlled vivariums (Pomatto et al, 2020) (Fig. 4A). The MSC in *Nrf2* null mice, which are considerably more fragile than in wild-type mice, may influence individual animal survival (Dodson et al, 2021). It is not clear that MSC in each tissue produce transit-amplifying progenitor cells (TA) as occurs in the intestinal crypt, where NRF2 engages in fine tuning of cell lineages (Yagishita et al, 2021).

Moreover, due to the proliferative role of TA, NRF2-signaling may play a significant role for other cells to proceed toward normal differentiation and to avoid impaired differentiation, including cancer stem cell production. The fact that NRF2 in cancer cells can acquire point mutations at multiple sites for gain of stability in its functional domains (*e.g.*, interfaces to KEAP1 or β−TRCP), such gain of function by mutated *NRF2* contributes to the survival of abnormal cells and finally, tumor/cancer following input from additional oncogenic pressures. Since cancer stem cells, which mimic MSCs, utilize NRF2 signaling to survive, the fine-tuning of NRF2 function might be important for the maintenance of a healthy MSC niche.

Because *Nrf2* null mice survive after birth with the functional establishment of each tissue, the absence of NRF2 signaling in such MSCs may be compensated by other transcription factors, at least to some degree. Canonical NRF2 target genes utilize AREs (Liu et al, 2019) shared with other CNC factors (especially, NRF1, NRF3, or NFE2p45 in erythroid cells). A reverse compensation can also happen. Indeed, *Nrf1* null mice die at embryonic day (ED) 13.5 due to hepatocyte dysfunction and impairment of early hematopoietic development (Chan et al, 1998).

In contrast to this late embryonic lethality in *Nrf1*-deficient mice, compound *Nrf1* and *Nrf2* knockout mice die at ED 9.5 exhibiting extensive apoptosis (Leung et al, 2003). Although this phenotype emerges earlier in embryonic development, it is notable that NRF2 functioned on the NRF1 dominant-ARE gene expression machinery to prolong animal longevity by 4 days. Interestingly, complete knockout mice of *sMafs*, which are heterodimeric partner molecules of both NRF1 and NRF2, also die at ED 13.5 (Yamazaki et al, 2012), as do knockout mice of Jun proto-oncogene (*c-Jun*), which possibly binds to AREs (Eferl et al, 1999).

Knockouts of *Trx2*, *Gss*, *TrxR1*, *Notch1*, and *Gclc*, which are ARE-containing genes regulated by NRF2 and NRF1, show embryonic lethality as well (Bondareva et al, 2007; Dalton et al, 2000; Nonn et al, 2003; Swiatek et al, 1994; Winkler et al, 2011). Although *Nqo1* knockout mice do not show a lethal phenotype (Diaz-Ruiz et al, 2019), several types of loss-of-function mice with NRF2 target genes experience mortality earlier than *Nrf1* null mice. Therefore, NRF2 might compensate for the loss of expression of these genes until ED 13.5 in the *Nrf1* null mice (Fig. 4A). In some cases, ARE sequences include TPA (12-O-Tetradecanoylphorbol-13-Acetate)-responsive element (TRE) or cAMP (cyclic adenosine monophosphate) response elements (CRE) sites, which are recognition sequences for transcription factor AP-1 (Jun proto-oncogene [JUN] and Fos proto-oncogene [FOS] family) (Jochum et al, 2001) or the CREB/ATF (cAMP-responsive element binding protein/activating transcription factor) family (Hai and Hartman, 2001), respectively (Fig. 4B).

This overlap might account for a lack of embryo lethality in *Nrf2* null mice. However, many toxicological studies have shown that the *Nrf2* null condition in whole body or in cell-specific constructs exhibit increased acute sensitivity to toxins and altered susceptibility to tumorigenesis. They do not develop spontaneous tumors. However, these compensatory factors do not provide a full fail-safe mechanism for specific ARE gene expression driven by NRF2-sMAF for maintenance of long-term development and survival.

Given that therapeutic approaches directed at stem cells are developing recently, along with the characterization of stem cells that are in perpetual self-renewal or able to differentiate into specialized somatic cell types, the identification and classification of ARE genes regulated by each CNC or related transcription factor in both MSC and niche somatic cells should be delineated at the *in vivo* level. Also, elucidation of the details by which cell-specific cases, conditions, and timing affect the contributions of NRF2 are needed.

## LACHESIS: Cell Differentiation and Proliferation

### Cell differentiation

**FIG. 5.**
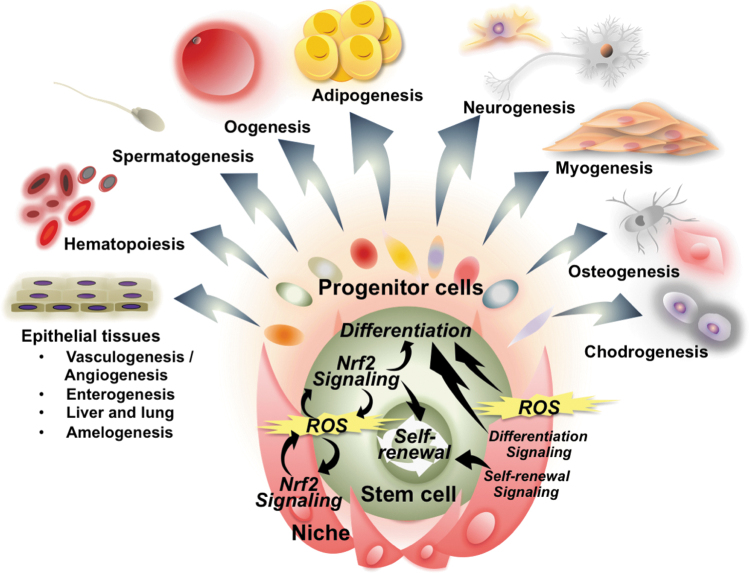
**NRF2 in multipotent stem cells and niche cells influence cell differentiation in multiple types of tissues.** The niche is the specific microenvironment surrounding stem cells that sustains them. NRF2 signaling expressed in the stem-niche unit, including multipotent stem cells (tissue-specific stem cells) and niche somatic cells, may facilitate maintenance of stem cells, self-renewal, and transition of stem cell fate (differentiation) in multiple types of tissues. A potential underlying molecular mechanism is the modification of physiological ROS signaling by NRF2-mediated redox regulation in stem/niche cells. Further, specific target genes that are uniquely expressed in the stem/niche unit in each tissue may be regulated by the NRF2-ARE machinery, resulting in the facilitation of cell differentiation processes. ROS, reactive oxygen species.

*Nrf2* null mice are viable, fertile and exhibit an outwardly normal phenotype, indicating that NRF2 is dispensable for development. However, recent studies suggest that subtle changes in phenotype reveal effects on tissue development, including cellular differentiation, some of which are mediated by disturbed crosstalk between NRF2 and other transcription factors (Fig. 5). Key observations from these mice are summarized later, beginning with epithelial tissue development and cell differentiation (*vasculogenesis/angiogenesis*, *enterogenesis*, *liver and lung*, and *amelogenesis*).

#### Vasculogenesis/angiogenesis

A congenital intrahepatic shunt, not seen in wild-type mice, was observed in *Nrf2*-deficient mice with a high prevalence (∼66%). This shunt directly connected the portal vein to the inferior vena cava and displayed characteristics of a patent ductus venosus (Skoko et al, 2014). A similar phenotype was expressed in both aryl hydrocarbon receptor (*Ahr*) and aryl hydrocarbon receptor nuclear translocator (*Arnt*) deficient mice in all animals (Lahvis et al, 2000; Walisser et al, 2004). A reciprocal NRF2-AhR gene expression system driven by AREs/XREs on each gene's functional promoter region might reflect signaling crosstalk (Miao et al, 2005; Shin et al, 2007), revealing a common phenotype. In neural tissues, such as retina or cerebrum, reduced NRF2 function leads to impaired vascular formation (Li et al, 2016; Uno et al, 2010; Wei et al, 2013).

However, in a mouse model of pregnancy-associated hypertension, *Nrf2* deficiency decreased the rate of perinatal morbidity (Nezu et al, 2017). The HIFs target genes are regulated by hypoxia response elements (HREs). The contribution of HIFs in vasculogenesis through VEGF signaling has been noted (Li et al, 2016). The HIFs (HIF1α and HIF2α) and their heterodimeric partner ARNT (HIF1β), which is also an AhR heterodimeric molecule, could regulate *Vegf* and heme oxygenase-1 (*Hmox1*) gene expression. Expression of these genes is also controlled by NRF2-ARE machinery. It might be that NRF2-HIF crosstalk contributes to vasculogenesis/angiogenesis through ARE/HRE signaling.

#### Enterogenesis

The small intestine has relatively high levels of *Nrf2*-expression (https://www.ncbi.nlm.nih.gov/gene/18024) where canonical NRF2 signaling contributes to host defense from exogenous toxicants and microbiota products. The small intestine is one of the fastest self-renewing tissues in the body. The proliferation of MSCs produces TA that turn into specialized epithelial cell types of absorptive (enterocyte) and secretory (goblet, Paneth and enteroendocrine) functions. NOTCH and its downstream effectors are pivotal for the maintenance of stem cells, guiding the proliferation of progenitor cells and the balance of differentiation toward different cell lineages.

Genetic (and pharmacologic) activation of NRF2 has been shown to perturb the dialog of the NOTCH cascade through negative regulation of *Math1* in progenitor cells, leading to enhanced enterogenesis (Yagishita et al, 2021). *Keap1^F/F^::Villin-Cre* mice, which exhibit enhanced NRF2 activation in the small intestinal epithelium, showed longer intestines and taller villus heights. The phenotype was cancelled in *Keap1^F/F^::Nrf2^F/F^::Villin-Cre* mice. Multiple ARE sequences lie on the *Math1* promoter/enhancer region, which can also function with other CNC transcription factors such as NRF1, NRF3, and BTB and CNC homology basic leucine zipper transcription factor (BACH)1. Thus, NRF2 can affect the differentiation plasticity of the intestinal epithelium.

#### Liver and lung

The association between hepatic epitheliogenesis and NRF2 has been demonstrated using mouse models. Bellanti et al (2021) demonstrated that inhibition of NRF2 stimulates differentiation of liver stem and progenitor cells (hepatoblasts) to hepatocytes/cholangiocytes. They employed a xenograft model that underwent transplantation of hepatic progenitor-like cells treated by the chemical compound ARE expression modulator 1 (AEM1), which is reported to inhibit the NRF2 downstream effect without altering *Nrf2* expression. *Nrf2* suppression accelerated recovery after liver injury, where it was observed that the damaged parenchyma was replaced with *Nrf2*-suppressed transplanted cells that harbor distinctive differentiated cells. Cholangiocytes are one of the hepatic epithelial cells that line the intrahepatic and extrahepatic bile ducts. It was shown that liver-specific activation of *Nrf2* in *Pten* (phosphatase and tensin homolog deleted from chromosome 10) knockout background mice displayed severe hepatomegaly. The livers in this model mouse showed abnormal expansion of ductal structures consisting of cholangiocytes (Taguchi et al, 2014). Since the functional loss of PTEN increases AKT phosphorylation, which promotes cell growth and proliferation, the crosstalk between NRF2 and PTEN was suggested as an associated molecular mechanism.

Cell differentiation of alveolar epithelial type II cells is developmentally induced in the fetal lung along with upregulated synthesis of an immune modulator, surfactant protein A (SP-A), that protects the alveolar epithelium from exposure to high O_2_ tension and inhaled pathogens after birth. Mishra et al (2021) reported that NRF2 transcriptionally regulates SP-A and other immune modulator genes during the cell differentiation process of human fetal lung epithelial cells. In mouse fetal lung, the expression of NRF2 and SP-A was elevated from 14.5 to 18.5 days post-coitum, suggesting that transcriptional regulation of SP-A by NRF2 may underlie the protective function of epithelium in fetal lung.

#### Amelogenesis

The incisors of rodents contain an iron-rich enamel that grows throughout life. There is one obvious phenotype in adult *Nrf2* null mice: The maxillary incisors are decolorized and become grayish white. There is less iron on the surface of *Nrf2* null incisors due to aberrant deposition of iron into the papillary layers during the late maturation stage of ameloblasts. Consequently, ameloblasts proceed to degenerative atrophy. Reduced ferritin gene expression (under the regulation of NRF2/ARE) likely influences the observed iron transport defect in the enamel of *Nrf2* null mice (Yanagawa et al, 2004).

Recently, mice with autophagy-related genes *Atg7*, *Atg5*, or *Sqstm1* (*p62*) deleted specifically in ameloblasts were reported; deletion of *Atg5* and *Atg7* (but not *p62*) leads to aberrantly decolorized incisors (Sukseree et al, 2020). Although NRF2 regulates the expression of these genes (Pajares et al, 2016), the basis for the partial association between NRF2 and autophagy in ameloblast development in enamel is unclear.

#### Folliculogenesis/oogenesis/placentation

Oxidative stress is associated with normal ovarian aging (Hamatani et al, 2004; Lim and Luderer, 2011; Tarin, 1996) and deletion of *Gclm*, an NRF2 target gene and subunit of the rate-limiting enzyme in glutathione (GSH) synthesis, causes accelerated ovarian aging (Lim et al, 2015a). Comparison of the numbers of ovarian follicles in wild-type and *Nrf2* null mice shows no difference at 35 days of age. However, by 10–12 months of age, the remaining primordial follicle pool is significantly smaller in *Nrf2* null mice along with a reduction of ovarian GSH levels (Lim et al, 2015b).

NRF2 is detected in oocytes and granulosa cells, but its expression is reduced in aged mice. *Nrf2* knockdown by injection of *Nrf2* small interfering RNA (siRNA) into fully grown oocytes showed that NRF2 contributed to the formation/stability of the meiotic spindle and the regulation of meiotic progression; *Nrf2* knockdown was associated with interference of *CyclinB1*/*Cdk 1* expression, thereby implicating a role for NRF2 in meiotic division. NRF2 overexpression ameliorated maternal age-associated oocyte meiotic defects (Ma et al, 2018).

The establishment of a placenta is critical for embryonic development and a successful pregnancy outcome. On ED 18.5 the fetal weight of the *Nrf2* null mouse was significantly reduced *versus* wild-type, indicating a decrease in placental efficiency (birth weight: placental weight). Reductions in both total and labyrinth-volume in the placenta of *Nrf2* null mice were observed (Kweider et al, 2017).

Interestingly, NRF2 transcripts were upregulated during syncytiotrophoblast differentiation from cytiotrophoblasts, and they dramatically reduced by hypoxia in the human pre-eclampic placenta. Moreover, *NRF2* knockdown experiments in cytotrophoblasts showed an inhibitory effect for induction of one microRNA (miRNA), *miR-1246*, whose direct targets include *GSK3*β and *AXIN2* (Chai et al, 2016), *CYP19A1* (P450 Aromatase) as well as *CEBP*β and peroxisome proliferator-activated receptor γ (*PPAR*γ), all implicated as key factors for placental differentiation (Barak et al, 1999; Toda et al, 1996) and as direct NRF2 target genes (Hou et al, 2012; Kim et al, 2018a). Both miR-1246 and the *CYP19A1* promoter/enhancer bear functional AREs (Muralimanoharan et al, 2018), raising the possibility of NRF2-ARE signaling between maternal tissues and the embryo in embryogenesis. The role of NRF2 in placentation should be elucidated in *in vivo* studies.

#### Spermatogenesis

Spermatozoa are one of the most susceptible cells to oxidative damage due to large amount of polyunsaturated fatty acids in their cell membranes, which can be oxidized by ROS. Low levels of ROS are necessary for sperm capacitation, the process by which sperm become capable of fertilizing an oocyte, and for the acrosome reaction, which enables the sperm to penetrate the zona pellucida of the oocyte and fuse with its membrane (de Lamirande et al, 1997). In a rodent model, the decline in male fertility with aging is associated with increasing oxidative damage and decreased antioxidant capacity in the male reproductive system (Weir and Robaire, 2007).

Indeed, vacuolization of seminiferous tubules, decreased testicular weights, decreased testicular and epididymal sperm counts, and decreased sperm motility were observed with decreased testicular and epididymal antioxidant capacity in aged-*Nrf2* null mice from 2- to 4-month-old (Nakamura et al, 2010). In humans, a strong association between functional polymorphisms in the *NRF2* promoter and seminal plasma superoxide dismutase (SOD) activity with the risk of oligoasthenoospermia (reduced sperm motility and count) has been described (Chen et al, 2012a; Yu et al, 2012). A reduction of *NRF2* promoter activity might affect direct target gene expression.

#### Osteogenesis

Bone tissue receives many inputs from the endocrine, nervous, and immune systems, and it responds with calcium metabolism, mechanotransduction, and hematopoiesis (Harada and Rodan, 2003). *Nrf2* null mice have a lower bone mass featuring lower bone mineral density. The bone strength of femurs and vertebral bodies were observed to be significantly lower in comparison to wild-type controls (Ibanez et al, 2014; Kim et al, 2014; Park et al, 2014; Sun et al, 2015). These data demonstrated that NRF2 plays an important role in bone homeostasis and mechanotransduction.

ATF4 is a transcription factor that regulates differentiation of osteoblast and bone mass by possibly activating AREs. Interestingly, a similar but more severe phenotype was observed in *Atf4* null mice, which exhibit low viability, with delayed bone formation during embryonic development and low bone mass throughout postnatal life (Yang et al, 2004). Further, *Atf4* null mice exhibited a reduction in oxidative stress-induced gene expression, resistance to oxidative death, and decreased consumption of GSH. The direct heterodimer formation between ATF4 and NRF2 was observed at the cellular level on the *Hmox1* enhancer (He et al, 2001) and recently, NRF2-ATF4 crosstalk has been considered for maintenance of mitochondrial quality (Kasai et al, 2019). Both factors might be able to sense and react to stress in the cells comprising bone tissue.

There are two major cells for maintaining bone metabolism and development: osteoblasts derived from mesenchymal stem cells and osteocytes along with osteoclasts derived from the monocyte/macrophage hematopoietic lineage. Osteoblasts and osteocytes work together to remove old bone and add new bone. NRF2-overexpressing osteoblastic MC3T3-E1 cells are blocked from ongoing differentiation toward maturation without affecting either cell survival or gene expression of several master regulators required for differentiation in cultured osteoblastic cells.

A likely mechanism considers interference with RUNX2-dependent transcriptional activation by direct interaction between NRF2 and RUNX2, a master gene of osteoblastogenesis (Hinoi et al, 2006). With osteoclastogenesis it was reported that activation of canonical NRF2-signaling seemed to suppress RANKL induced osteoclast differentiation *via* intracellular ROS attenuation in a cell culture differentiation system (Hyeon et al, 2013; Xue et al, 2019).

*In vivo* evidence is provided by NEKO mice in which NRF2 signaling is highly activated throughout the body due to *Keap1* gene disruption, except for upper digestive tract (esophagus) and skin in which *Nrf2* is selectively deleted (Yoshida et al, 2018). In NEKO mice, hypoplasia of bone mass was observed along with nephrogenic diabetes insipidus. Further, differentiation of both osteoclasts and osteoblasts was attenuated in *in vitro* differentiation experiments using primary cells derived from *Keap1*-null mice, suggesting association between constitutive activation of NRF2 and its impact on osteogenesis. Interestingly, in humans, inborn *de novo* mutations of *NRF2* have been identified leading to NRF2 accumulation and producing multisystem disorders, including mild developmental delays, short stature, and delayed bone age (Huppke et al, 2017). The mimicked phenotypes in mouse and human further imply that NRF2-signaling contributes to maintenance of bone homeostasis.

In summary, accumulating evidence, including *in vivo*, *in vitro*, and clinical observations, indicates association between NRF2 signaling and osteogenesis. NRF2 signaling might be a therapeutic target for bone metabolism-related diseases, such as osteoporosis. However, since bone metabolism involves multiple factors, including mechanical stimuli that activate bone remodeling, age, and sex, further studies involving dynamics of bone biology are required.

#### Chondrogenesis

*Nrf2* transcripts in tibia were detected at ED 15.5 in both proliferating and pre-hypertrophic chondrocytes and expressed in all chondrocytes by postnatal day 1. Forced expression of NRF2 markedly inhibited *in vitro* chondrogenesis in mouse pre-chondrogenic ATDC5 cells (Hinoi et al, 2007). In human T/C28a2 chondrocytic cells, lower NRF2 signaling leads to their apoptosis under shear stress (Healy et al, 2005). It seemed that NRF2 signaling contributed to cartilage formation in T/C28a2 cells. Chondrogenesis could be controlled by both overexpression and downregulation of NRF2 signaling, a paradox possibly explained by taking into consideration the concept that appropriate expression of NRF2 signaling is required for normal chondrogenesis.

#### Myogenesis

Impaired skeletal muscle regeneration in *Nrf2* null mice using an ischemia injury model was observed wherein it was noted that NRF2 regulated the expression of myogenic regulatory factors. The 5′-proximal promoter region of the transcription factors, myogenic differentiation 1 (*Myod1*) and Myogenin (*Myog*), contains highly conserved functional ARE sequences among various animals from fish to human (Al-Sawaf et al, 2014). Using skeletal muscle-specific stem cells or C2C12 myoblast cells, it was concluded that *Myod1* is a direct NRF2 target gene in postnatal satellite cells, whereas for *Myog* promoter activity, NRF2 acts as a suppressor (Rudnicki et al, 2008; Zammit, 2017).

NRF2 contributes to satellite cell proliferation by upregulation of *Myod1* gene and concurrently suppresses their differentiation to myotubes by downregulation of *Myog* expression. However, a contrasting view was also reported in which *Nrf2* null mice did not show any defect in skeletal muscle regeneration in an acute muscle damage model using cardiotoxin. NRF2 inhibited *Myod1* and *Myog* expression in proliferating myoblasts (Yamaguchi et al, 2015). Kageyama et al (2007) confirmed an existing NOTCH-NRF2 axis through HES expression. This interaction is mechanistically conceivable through possible negative regulation of their downstream expression due to nonfunctional hetero-dimer formation (HES factor with MYOD1/MYOG bHLH-transcription factor). It has also been reported that excess NRF2 signaling evoked reductive conditions that hampered differentiation of muscle satellite cells (Rajasekaran et al, 2020). Although further studies are necessary to reveal the complete regulation of myogenic genes by NRF2, many data already provide evidence that the function of NRF2 is strongly dependent on the state of cell differentiation and mode and magnitude of injury.

#### Hematopoiesis

Many studies have revealed that high levels of ROS influence stem cell function. Although postnatal hematopoietic stem/progenitor cells (HSPCs) are susceptible to oxidative stress, the basal ROS levels of HSPC in *Nrf2* null mice are not elevated. Thus, homeostatic ROS is not always deleterious to HSPC survival. Instead of dysfunctional ROS metabolism by impaired NRF2 signaling, global defects of cytokine signaling were posited as the main reason for abnormal HSPCs in *Nrf2* null mice (Merchant et al, 2011).

Although NRF2 has been previously reported to be dispensable for erythropoiesis and megakaryocyte differentiation (Chan et al, 1996; Kuroha et al, 1998; Motohashi et al, 2010), Tsai et al (2013) conducted cell-lineage sorting for *Nrf2* null mice. They reported that loss of functional NRF2 leads to expansion of the hematopoietic progenitor pool (at short-term HSPC and continued through multipotent progenitor stage) but spared the most primitive long-term HSPCs. Moreover, through a transplant experiment, it was suggested that NRF2 regulates proliferation and differentiation of HSPC in a cell-intrinsic manner.

These results imply that NRF2 has a critical role in maintaining HSPC function through quiescence and self-renewal and by extension, differentiation. It was also recognized from *Nrf2* loss-of-function mice that NRF2 does not control lineage specification in hematopoiesis unlike other CNC family genes such as *Nfe2p45* (Kuroha et al, 1998; Motohashi et al, 2010) or *Bach2* (Muto et al, 1998).

It was reported that *Keap1* deficiency in HSPC enhances granulocyte-monocyte differentiation. It seemed that NRF2 activation in HSPC promoted the differential preference for this lineage due to elimination of this phenotype when *Nrf2* was deleted from *Keap1* null HSPC (Murakami et al, 2014). However, *Nrf2* null HSPC did not show an altered preference for cell lineage commitment. Maybe this phenotype arises from combined disturbance of NRF2 crosstalk with other proteins interacting with the KEAP1 degron, such as IKKB (Bottero et al, 2006; Kim et al, 2010; Lee et al, 2009). Nonetheless, NRF2 signaling influences cell fate determination in hematopoiesis.

In aged *Nrf2* null mice, abnormal red cell morphologies such as Howell–Jolly bodies, schistocytes, and acantocytes indicative of hemolytic anemia were observed. However, young mice are not anemic. Other factors such as NFE2p45, NRF1, NRF3, and cJUN could compensate for ARE gene expression or another pathway could counterbalance within the *Nrf2* null milieu. Consequently, disruption of *Nrf2* causes regenerative immune-mediated hemolytic anemia associated with splenomegaly by age-dependent increases of oxidative damage (Lee et al, 2004).

A similar disturbance might be observed in megakaryopoiesis, due to the diverse functional roles of the ARE/MARE (MAF homodimer specific binding sites) with NF-E2p45 (Motohashi et al, 2010). The hidden phenotype from loss of function of NRF2 signaling seems to be dependent on stress intensity/amount of ROS.

The association between NRF2 activity and lymphocyte differentiation and maturation has also been studied. T-cell-specific NRF2 activation mice demonstrated an increased number of CD25^+^Foxp3^+^ regulatory T cells (Tregs) and a decreased number of CD11b^+^CD11c^+^F4/80^+^ macrophages in kidneys, indicating a direct association between NRF2 and lymphocyte differentiation (Noel et al, 2015). The altered cell composition of lymphocytes provoked by NRF2 activation appears to underlie notable tissue protection observed in acute kidney injury model mice.

In another line of study, it was reported that sulforaphane, a potent inducer of NRF2, significantly reduced the differentiation of lipopolysaccharide-stimulated murine splenocytes into plasma B cells and germinal-center B cells (Moon et al, 2021). Due to the broad range of targeted pathways by sulforaphane, the dependency of NRF2 underlying the reported observation is not clear. However, inhibition of B-cell differentiation evoked by sulforaphane may explain the anti-arthritic effect found in the collagen-induced arthritis model mice treated with sulforaphane.

#### Neurogenesis

Due to its high content of unsaturated fatty acids and transition metals and high utilization of oxygen, the brain is known to generate large amounts of ROS. A global brain ischemia model employing both *Nrf2* null and control mice demonstrated that NRF2 contributed to endogenous neurogenesis, especially in the proliferative stage of neural stem/progenitor cells (NPCs). Forced expression of NRF2 restored NPC proliferation, differentiation, and viability that was impeded by amyloid β (Aβ), a toxic peptide believed to cause synaptic dysfunction and neuronal loss in Alzheimer's Disease, the major dementia disorder (Karkkainen et al, 2014).

Moreover, Aβ reduced differentiation of neurons from *Nrf2*-deficient NPCs. Interestingly, B-site amyloid precursor protein cleaving enzyme 1 (BACE1), which is a rate-limiting enzyme for producing Aβ, was reported as an NRF2 regulated gene in both mice and humans (Bahn et al, 2019). The fact that NRF2 is protective against neurodegeneration is well established both in cell cultures of primary postmitotic neurons and in animal models (Kanninen et al, 2008; La Rosa et al, 2019; Shih et al, 2003). The conclusion is supported by a study using NPC isolated from the subgranular zone in the brains of postnatal or 3-month-old *Nrf2* null and control mice, and by knockdown of *Nrf2* expression in wild-type NPC (Robledinos-Anton et al, 2017).

#### Adipogenesis

Adipogenesis is a highly regulated process at the transcriptional level (Lee et al, 2019), and the potential crosstalk of NRF2 with major adipogenesis transcription factors (PPARγ, CCAAT/enhancer-binding protein-β [CEBPβ]) has been studied. Several *in vitro* studies have shown that the NRF2 pathway affects adipogenesis. *Nrf2* knockout mice have lower amounts of adipose tissue and smaller adipocytes, and *in vitro* differentiation of *Nrf2*-deficient MEF showed impaired adipogenesis (Pi et al, 2010). Silencing of *Nrf2* in 3T3L1 preadipocytes partially impaired their potential to differentiate into mature adipocytes, whereas the activation of the NRF2 pathway by knocking down *Keap1* led to improved adipocyte differentiation.

Activation of the *Ppar*γ promoter by NRF2 has been shown in these cells by ChIP and luciferase experiments (Pi et al, 2010). Similarly, the deletion of *Nrf2* in adipocytes in mice of the *ob/ob* background driven by *Fabp4/aP2* promoter led to lower adipose tissue mass (Xue et al, 2013). However, adipocyte-specific deletion of *Nrf2* driven by the *Adipoq* promoter did not lead to a significant difference in fat mass or in adipocyte size in mice exposed to high-fat diet (Chartoumpekis et al, 2018); discrepancies were possibly attributed to the different knockout strategies of *Nrf2*.

Deletion of *Nrf2* in all tissues may lead to differences in adipocytes that are dependent on neighboring tissues or in other secreted factors (hormones, growth factors) that remain to be elucidated. The adipocyte-specific deletion of *Nrf2* is the most appropriate strategy to investigate the effect of NRF2 in adipogenesis *in vivo*. The use of *Fabp4/aP2* promoter, historically the most popular strategy to generate adipocyte-specific knockout mice, has been shown to target brain, muscle, and macrophages as well, whereas *Adipoq* appears to be a more adipocyte-specific promoter (Mullican et al, 2013).

Employing a more adipocyte-specific promoter for *Nrf2* deletion does not seem to affect adipocyte differentiation or mass. The *in vitro* observations that NRF2 induces *Ppar*γ promoter directly (Pi et al, 2010) or represses *Ppar*γ through activation of AhR (Shin et al, 2007), and can induce *Cebp*β (Hou et al, 2012) provide evidence for molecular interactions that appear not to be translated into a phenotype in *in vivo* models. Likely, the impact of abrogated NRF2 signaling *in vivo* is well compensated.

The whole-body *Nrf2* deletion appeared to partially protect from high-fat diet-induced obesity in mice and led to a more insulin-sensitive phenotype whereas adipocyte-specific deletion of *Nrf2* led to worse glucose tolerance without affecting body mass. The increased energy expenditure in whole body *Nrf2* knockout mice (Meakin et al, 2014; Schneider et al, 2016; Sun et al, 2020) is one of the mechanisms for amelioration of the metabolic phenotype, whereas adipocyte-specific deletion has no effect on energy consumption (Chartoumpekis et al, 2018).

Direct transcriptional regulation of adipogenesis factors by NRF2 provide important findings to understand the biological significance of NRF2 in adipogenesis. To decipher not only the role of NRF2 in adipogenesis that is regulated by transcriptional regulation in adipocytes, but also a neighboring microenvironment that includes endothelial cells, immune cells, and mural cells, cell heterogeneity and functional diversity of adipose tissue must be taken into consideration, where more precise approaches including a single-cell level of studies may be required.

### Cell proliferation

Most normal cells in adult animals are at least transiently arrested in the G0 state of the cell cycle, proliferating as needed to replace cells that have been lost by tissue injury and/or cell death. On the other hand, cancer cells actively proliferate. Consequently, the role of NRF2 signaling and cell proliferation has been studied principally using tissue damage or cancer models.

#### ROS-dependent mechanism and NRF2

One of the first studies to describe an ROS-dependent mechanism for NRF2 in proliferation in non-cancer cells was performed in mice undergoing partial hepatectomy. Mice lacking *Nrf2* showed delayed proliferation of hepatocytes in the regenerating liver, an effect attributed to increased oxidative stress, leading to impaired Insulin/IGF1 signaling (Beyer et al, 2008). In another study using primary alveolar type II cells from the lungs of wild-type and *Nrf2* knockout mice, it was shown that proliferation was significantly slower in the *Nrf2*-disrupted cells (Reddy et al, 2007). *Nrf2* knockout cells also exhibited increased ROS levels and decreased GSH levels. Treating these cells with N-acetyl cysteine reduced ROS levels but did not restore cellular proliferation completely, whereas treatment with GSH reduced ROS and rescued cell proliferation to wild-type levels. A similar observation has been made in human glioblastoma cell lines, where the silencing of *NRF2* resulted in decreased proliferation that was rescued only with GSH supplementation and not N-acetyl cysteine despite the fact that both treatments reduced ROS levels (Jia et al, 2017). A mechanism involving ROS elimination by oncogene-induced NRF2 expression has been shown to affect cell proliferation.

The MEFs endogenously expressing the oncogene *Kras* showed decreased ROS levels and increased NRF2 signaling dependent on the RAF-MEK-ERK-JUN pathway. This was also verified in mouse models of pancreatic and lung cancer that expressed these oncogenes and exhibited increased *Nqo1* expression (a prototypical NRF2 target gene) and reduced oxidative stress, as evidenced by low 7,8-Dihydro-8-oxo-2′-deoxyguanosine (8-oxo-dGuo) levels (DeNicola et al, 2011). Loss of *Nrf2* led to less proliferation of cells *in vivo* and *in vitro*; this proliferation defect was rescued by treatment with N-acetyl cysteine, indicating that ROS levels were the driving factor in this model.

In esophageal cancer patients, NRF2 expression and downstream signaling has been found to be elevated compared with non-cancer tissue from the same subject and associated with worse clinical outcomes (Kitano et al, 2018). By employing esophageal cancer cell lines in the same study, *NRF2* deletion was shown to decrease cell proliferation and increase ROS levels, which led to increased p38 MAPK signaling and decreased expression of *CYCLIND1*. However, no rescue experiment was performed by treating *NRF2*-disrupted cells with N-acetyl cysteine so as to assess possible dependency on ROS.

Treatment of pancreatic carcinoma cell lines with media from pancreatic stellate cells enhanced cell proliferation in an NRF2-dependent manner; ROS levels were decreased in the treated cells. However, no rescue experiment by, for instance, increasing ROS was performed and it is possible that other NRF2-dependent mechanisms may affect cell proliferation such as changes in metabolic program (Wu et al, 2016).

#### Signaling crosstalk-dependent mechanism and NRF2

Crosstalk between NRF2 and NOTCH1 was one of the first direct interactions of NRF2 with other factors to be described that affects the proliferation of cells. *Notch1* is an NRF2 target gene possessing AREs in its regulatory region. Disruption of *Nrf2* leads to decreased expression of NOTCH1 and its target genes in MEFs and in the liver. *Nrf2* knockout mice show dampened proliferation of hepatocytes after partial hepatectomy, which is rescued by genetic overexpression of the NOTCH1 intracellular domain (Wakabayashi et al, 2010).

This NRF2-NOTCH axis has been shown to affect cell proliferation in breast cancer cell lines (Zhang et al, 2019); NRF2 overexpression induced NOTCH signaling and cell proliferation whereas inhibition of NOTCH signaling by a γ-secretase inhibitor (DAPT) abrogated this phenotype. NRF2 pathway activation or disruption has been shown to increase or decrease NOTCH signaling and cell proliferation, respectively, in both oral squamous cell carcinoma cell lines and in normal murine tongue (Fan et al, 2017).

From a different perspective, NRF2 signaling mediated hepatocyte proliferation was reported (Shirasaki et al, 2014). Shirasaki et al tested the contribution of NRF2 signaling to compensatory hypertrophy using a portal vein branch ligation technique. In the non-ligated liver lobes, NRF2 activation and *Nrf2* knockout accelerated and diminished compensatory hypertrophy. These observations were supported by corresponding measures of cell proliferation. The underlining molecular mechanism is likely to be related to long-lasting GSK3 phosphorylation, which is reported to enhance the nuclear accumulation of NRF2, as observed in the non-ligated liver lobes of NRF2 activation mice.

The interaction of p63 and NRF2 in the nucleus augments transcriptional activation and has been shown to enhance keratinocyte proliferation as it induces the cell cycle gene *CDK12* using *in vitro* and *in vivo* models (Kurinna et al, 2021). This interaction is conserved in humans and mice and the induction of CDK12 it promotes is important, as treatment with the CDK12 inhibitor THZ531 reduced proliferation. This novel p63-NRF2 interaction is also important, because p63 is necessary for epithelial stratification (Koster et al, 2004) and is overexpressed in cancers (Graziano and De Laurenzi, 2011).

The crosstalk of NRF2 with *Myod* is another interesting example of how direct interaction of NRF2 with another pathway can have an effect on cell proliferation. *Nrf2* knockout mice showed delayed muscle regeneration after hind limb injury and reduced *Myod* expression, a major regulator of muscle regeneration (Al-Sawaf et al, 2014). *Myod* was found to be a direct target of NRF2 with a functional ARE in its regulatory region. Enhanced NRF2 signaling through silencing of *Keap1* led to increased proliferation of C2C12 myoblasts along with increased expression of *Myod*.

PTGR1, an oxidoreductase that is involved in the metabolism of eicosanoids and lipid peroxidation, is overexpressed in the early stages of hepatocarcinogenesis and regulated by NRF2 (Sanchez-Rodriguez et al, 2017). It is not completely clear how PTGR1 affects cell proliferation, but it appears to affect cancer cell proliferation in various cancers (Wang et al, 2021). An approach to unravel mechanisms underlying the NRF2-mediated cell proliferation in non-small-cell lung cancers was performed using three distinct cell lines: A549 that has a point mutation of *KEAP1* and a wild-type epidermal growth factor receptor (*EGFR*), PC-9 cells that have no mutation of *KEAP1* but an activating deletion in *EGFR*, and NCI-H292 cells that possess wild-type *KEAP1* and *EGFR* (Yamadori et al, 2012).

EGFR signaling was found to activate the NRF2 pathway through MAPK/ERK kinase-ERK signaling pathways and to increase cell proliferation. In these cells, use of PI3K inhibitors did not affect cell proliferation whereas tyrosine kinase inhibitors for EGFR and MEK1/2 did indicate that the latter pathways should mediate the induction of proliferation. The mechanisms underlying the interaction of NRF2 with these pathways are not yet well established.

#### Metabolic pathway and NRF2 in cancer cell proliferation

**FIG. 6.**
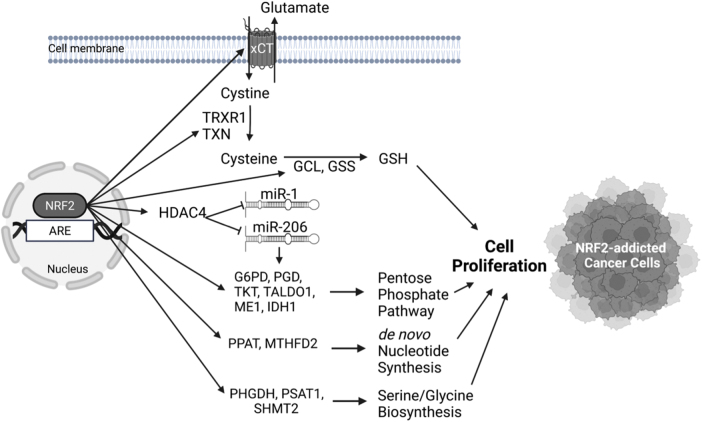
**NRF2 signaling affects cell proliferation in NRF2-addicted cancer cells *via* modification of cellular metabolism.** Many human cancer cells show persistent activation of NRF2 driven by dysregulating mechanisms, which partially contributes to the acceleration of cancer cell proliferation. One underlying mechanism is metabolic reprogramming directly or indirectly regulated by NRF2. The illustration was created with BioRender.com G6PD, glucose-6-phosphate dehydrogenase; GCL, glutamate cysteine ligase; GSH, glutathione; GSS, glutathione synthase; HDAC4, histone deacetylase inhibitor 4; IDH1, isocitrate dehydrogenase 1; ME1, malic enzyme 1; MTHFD2, methylenetetrahydrofolate dehydrogenase 2; PGD, 6-phosphogluconate dehydrogenase; PHGDH, phosphoglycerate dehydrogenase; PPAT, phosphoribosyl pyrophosphate amidotransferase; PSAT1, phosphoserine aminotransferase 1; SHMT2, serine hydroxymethyltransferase 2; TALDO1, transaldolase 1; TKT, transketolase; TRXR1, thioredoxin reductase 1; TXN, thioredoxin; xCT, cystine/glutamate antiporter (SLC7A11).

As a hallmark of cancer, metabolic reprograming is one of the major molecular events observed in cancer cells. Alteration of metabolism is required to support the increased energy required for continuous growth and rapid cell proliferation. Accumulating evidence shows an association between metabolic reprograming and aberrant activation of NRF2 in cancer cells (Fig. 6).

It has been shown that NRF2 regulates the expression of serine/glycine biosynthesis genes (*PHGDH*, *PSAT1*, *SHMT2*) *via* ATF4 in non-small-cell lung cancer cell lines and thus can support the proliferation of cancer cells (DeNicola et al, 2015). This finding is of clinical relevance as patients with lung tumors who showed high NRF2 expression also showed high ATF4 and serine/glycine biosynthesis gene expression and had decreased median survival (DeNicola et al, 2015).

Again, in lung cancer cell lines, NRF2 has been shown to accelerate proliferation by upregulating the expression of genes involved in the pentose phosphate pathway (*G6PD*, *PGD*, *TKT*, *TALDO1*, *ME1*, *IDH1*) through conserved AREs in their regulatory regions and in *de novo* nucleotide synthesis (*PPAT*, *MTHFD2*) (Mitsuishi et al, 2012). This leads to increased NADPH and nucleotide production and enhances the proliferation of cancer cells. It is important to note that the induction of these metabolic genes by NRF2 is also present in non-cancerous tissues such as in MEFs, forestomach, and intestine of mice that express lower KEAP1 levels (one *Keap1* allele knockout and one hypomorphic). Deletion of *Nrf2* in these mice abrogated these phenotypic changes. This proliferative effect of NRF2 in cancer and non-cancer cells requires the presence of active PI3K/AKT signaling, where it seems that activation of the PI3K/AKT pathway increases NRF2 pathway activation and vice versa, constituting positive feedback (Mitsuishi et al, 2012). The induction of pentose phosphate pathway genes and concomitant cell proliferation by NRF2 has also been described in A549 lung adenocarcinoma cell line *via* repression of miR-1 and miR-206 through histone deacetylase inhibitor 4 (HDAC4) (Singh et al, 2013).

NRF2 pathway hyperactivation in cancers has also been found to lead to increased accumulation of intracellular cysteine, thereby resulting in a glutamate-deficient state (Torrente and DeNicola, 2022). Specifically, NRF2 directly upregulates the expression of cystine/glutamate antiporter (xCT or SLC7A11) system (Sasaki et al, 2002). The increased intracellular cystine is converted to cysteine by the NRF2-dependent genes *TXN* and *TRXR1* by consuming NADPH. Two other NRF2 target genes *GCL* and *GSS* direct cysteine to the generation of GSH, which offer a survival and proliferation advantage to the cancer cells (Kang et al, 2019).

For this effect to take place, it is necessary for cysteine dioxygenase 1 (CDO1), an enzyme that catalyzes the irreversible conversion of cysteine to cysteine sulfinic acid, to be silenced, as it happens in human non-small-cell lung cancers that harbor mutations in *KEAP1*. This was shown by the use of primary MEFs from mice that harbor the *Keap1*R554Q mutation that leads to aberrant NRF2 pathway activation (Kang et al, 2019). The resulting glutamate-deficient state in lung cancers that bear *KEAP1* mutations was also highlighted in another study that employed a KRAS-driven lung cancer model. Loss of *Keap1* in these mice resulted in higher tumor burden with a higher proliferation rate that, in turn, was dependent on the conversion of glutamine to glutamate (Romero et al, 2017).

## ATROPOS: Cellular Senescence and Death

Cells in a terminal state undergo one of two additional fates: senescence or cell death, which are often triggered by similar biological signals. The molecular mechanisms underlying these alternative cell fate determinations are not fully understood. Excessive ROS concentrations can drive cells to the terminal stage of cell life, which highlights the role of NRF2 as an important player during the processes of cellular senescence and cell death *via* its redox regulatory actions. In addition to redox-dependent relevance, the interface between several signaling pathways in cellular senescence and cell death and NRF2 signaling has been verified.

### Cellular senescence

After undergoing a finite number of divisions, cells can enter into a permanent cell cycle arrest, termed cellular senescence. Senescent cells maintain their cell functions with phenotypic alterations, including alteration of metabolic activity and dramatic changes of gene expression (Herranz and Gil, 2018; Kumari and Jat, 2021). A wealth of data has demonstrated that cellular senescence and the process of aging and aging-related diseases have a tight association.

Studies using *Caenorhabditis elegans* and flies show that NRF2 influences their lifespans (Blackwell et al, 2015; Tsakiri et al, 2019). Moreover, livers of longer-lived rodent species, such as the naked mole-rat, show markedly higher levels of NRF2 signaling activity than shorter-lived rodents, which appears to be related to expression levels of KEAP1 and β-TCRP that target NRF2 for degradation (Lewis et al, 2015). Enhanced NRF2 signaling in a variety of slower aging animal models, such as genetic models (Snell dwarf mice), caloric restriction models, a rapamycin-mediated longevity model in *C. elegans*, was observed (Bruns et al, 2015).

A potential association between aging-mediated phenotypes and NRF2 activity is also demonstrated using *Nrf2* knockout mice (Hoshino et al, 2011). In addition, senescence limits the characteristics and functions of stem cells, which fundamentally affects age-related pathologies. For example, Anandhan et al (2021) demonstrated that NRF2 overexpression in neural stem progenitor cells in the subventricular zone of aging rats (11-month-old) exhibited improved behavioral function in comparison to control animals through improvements in neural stem progenitor cell proliferation, self-renewal, and neurogenesis. Although the detailed molecular mechanisms have been largely unexplored, these studies demonstrate that changes in NRF2 regulatory mechanisms with aging have an impact on age-related pathologies at a systemic level, as well as a cellular level.

Increasing ROS levels and accumulation of oxidative damage are major characteristics of aging, potentially leading to age-related pathologies. At low to modest doses, ROS are considered to be an essential biological cue for the regulation of normal physiological functions. Excessive levels of ROS, however, result in accumulated cell damage, which can lead to an acceleration of cell aging and cell death processes.

Thus, antioxidant networks to scavenge excessively produced ROS are critical to balance the production and scavenging of ROS to maintain cell homeostasis, a process wherein NRF2 plays a central role. It is interesting to note that an aging-mediated diminution of antioxidant enzymes is likely associated with declining NRF2 signaling (Zhang et al, 2015). In the aged *Nrf2* knockout mice (21- to 24-month-old), serum testosterone levels and testosterone production in Leydig cells were reduced significantly along with increased ROS levels in the testis compared with wild-type mice (Chen et al, 2015). The skin of *Nrf2* knockout mice showed accelerated photoaging phenotypes caused by ultraviolet B, which was related to elevated cutaneous oxidative stress levels and a significant decrease in cutaneous GSH levels (Hirota et al, 2011).

Bone marrow endothelial progenitor cells (EPCs) derived from young and aged mice demonstrated that the biological function of EPCs decreased with aging, wherein the expression levels of NRF2 and its target genes declined correspondingly (Wang et al, 2019). Further, *Nrf2* silencing impaired the function of EPCs and induced oxidative stress in EPCs from young mice, whereas NRF2 activation in EPCs from aged mice protected these cells against oxidative stress and ameliorated their biological dysfunction.

Collectively, the evidence suggests potential interconnections between NRF2-mediated redox regulation and the molecular physiology of cellular senescence. Beneficial effects by NRF2 activation for cell senescence have been suggested by many studies; however, it was also paradoxically demonstrated that NRF2 activation promotes senescence in fibroblasts. Hiebert et al (2018) showed that the skin fibroblasts derived from a genetic *Nrf2* activation mouse model as well as human skin fibroblasts treated with an NRF2 inducer showed senescence-associated phenotypes. It seems that increased ROS levels and subsequent DNA damage were not responsible for NRF2-mediated senescence, suggesting an existence of an alternative pathway.

The potential contributions of NRF2 in cellular senescence have been also examined with particular focus on signaling-mediated mechanisms. Chen et al demonstrated that mice with knockout of the 25-hydroxyvitamin D 1α-hydroxylase [1α(OH)ase] enzyme that generates an active form of vitamin D (1,25-dihydroxyvitamin D_3_) showed aging phenotypes, such as a shortened lifespan, an elevation of oxidative stress, and induced cell senescence. These phenotypes were rescued by supplementation with exogenous 1,25-dihydroxyvitamin D_3_ which binds to the vitamin D_3_ receptor (VDR) leading to a downstream pathway. Using MEFs, they demonstrated that the promoter region of the *NRF2* gene contains the predicted VDR binding site, which was activated by 1,25-dihydroxyvitamin D_3_ to induce *NRF2* expression. Their observations suggest an involvement of a transcriptional axis between VDR and *NRF2* to facilitate vitamin D mediated anti-aging and anti-cell senescence (Chen et al, 2019). Hutchinson-Gilford progeria syndrome (HGPS) is a rare fatal premature aging disorder, caused by a point mutation in the lamin A gene that produces a truncated mutant protein named progerin. Kubben et al (2016) reported that NRF2-ARE transcriptional activity was impaired in skin fibroblasts of HGPS patients. Further, they observed a PROGERIN-NRF2 direct interaction, which, in turn, caused mis-localization of NRF2 leading to impaired NRF2 transcriptional activity followed by elevated levels of oxidative stress and the progerin-induced aging phenotype. Recent studies reported a potential association between age-dependent alteration of miRNAs, such as miR-34a and miR-93, and NRF2 signaling in animal models (Csiszar et al, 2014; Li et al, 2011). Considering that some miRNAs regulate the NRF2 pathway by directly targeting NRF2, further study is encouraged to understand the new roles of NRF2 in aging processes involving miRNAs.

Sirtuin1 (SIRT1) has been reported to be involved in the regulation of cellular senescence and aging (Chen et al, 2020a), wherein the crosstalk between NRF2 and SIRT1 has also been studied by being linked to aging processes. A lower level of NRF2 protein was reported in oocytes from aged mice (Ma et al, 2018). *Sirt1* depletion reduced NRF2 expression in mouse oocytes, indicating the existence of a SIRT-NRF2 signaling crosstalk that can be associated with suppressed CYCLIN B1 expression and disrupted oocyte maturation.

Although several studies indicate an interaction between NRF2 and SIRT1-mediated cellular senescence using potential NRF2 inducers (Chen et al, 2020b; Kim et al, 2018b), further investigations employing genetic modification of *Nrf2* are required to dissect the direct contribution of NRF2 to SIRT1-mediated biological events that lead to cellular senescence.

### Cell death

**FIG. 7.**
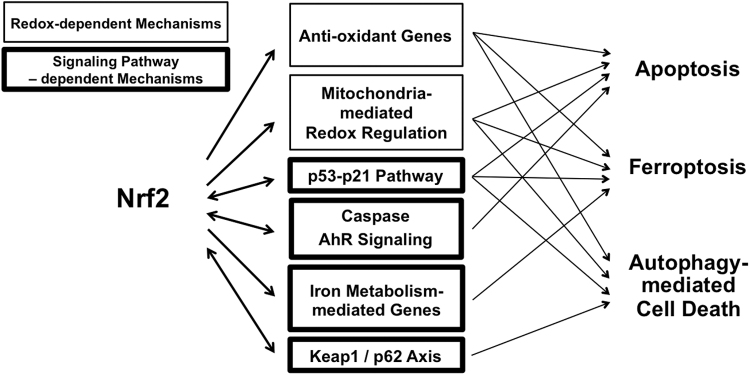
**Interaction between NRF2 signaling and regulated cell death.** Nrf2 regulates three types of regulated cell death: apoptosis, ferroptosis, and autophagy-mediated cell death *via* redox-mediated and signaling pathway-mediated mechanisms. AhR, aryl hydrocarbon receptor; p21, cyclin-dependent kinase inhibitor 1; p53, transformation related protein 53; p62, sequestosome 1.

The regulation of the balance between cell division and cell death is crucial for the development of organisms and the maintenance of biological homeostasis. Cells die from accidental cell death or regulated cell death. Regulated cell death includes several processes, such as apoptotic cell death, necroptosis, pyroptosis, ferroptosis, entotic cell death, necrotic cell death, parthanatosis, lysosome-dependent cell death, authophagy-dependent cell death, alkaliptosis, and oxeiptosis (Tang et al, 2019). A large body of evidence over the past decades has linked NRF2 signaling with multiple forms of regulated cell death. The strongest correlations are between NRF2 signaling and (1) apoptotic cell death, (2) autophagic cell death, and (3) ferroptosis (Fig. 7).

### Apoptotic cell death

The most commonly observed pathway of regulated cell death utilizes a caspase-dependent process, known as apoptosis. There are two types of apoptosis: physiologic apoptosis and pathologic apoptosis. Physiologic apoptosis is a critical molecular event in various circumstances, such as normal cell turnover, embryonic development, and wound-healing processes. Pathologic apoptosis, especially accelerated apoptosis, is often observed in immunodeficiency, neurodegenerative disease, and following ischemic damage, whereas inhibited apoptosis is found in cancers and autoimmunity. Here, giving most attention to pathologic apoptosis, potential associations between NRF2 signaling are summarized.

ROS is a central player in the regulation of the main pathways of apoptosis mediated by death receptors, the endoplasmic reticulum, and mitochondria. The tight association between NRF2-mediated redox regulation and apoptosis has been widely observed through loss-of-function approaches. In a streptozotocin-induced diabetes model, *Nrf2* knockout mice exhibited more severe diabetes than wild-type mice, and evoked severe cardiomyopathy along with increased apoptosis, accelerated inflammatory responses, and heightened oxidative stress in hearts (He and Ma, 2012).

*Nrf2* knockout mice exposed to cigarette smoke showed more extensive emphysema compared with wild-type mice along with increased oxidative stress, increased apoptosis, and pronounced inflammation. These observations were likely associated with nearly 50 NRF2-dependent antioxidant and cytoprotective genes whose expression in the lungs of *Nrf2* knockout mice were significantly lower than that of wild-type mice (Rangasamy et al, 2004).

*Nrf2* knockout mice used in a traumatic brain injury model displayed exacerbated brain injury as shown by increased oxidative stress markers, pro-inflammatory cytokines, and apoptosis markers (Bhowmick et al, 2019). The protective effects of NRF2 activation against pathophysiological apoptosis have been reported using several types of disease models, such as osteoarthritis, ischemia-reperfusion, and diabetic skin ulcers (Khan et al, 2018; Long et al, 2016; Meng et al, 2017), where neutralizing excess ROS levels by NRF2 mediated redox regulation seems to be one of the potential mechanisms.

Mitochondria are key organelles for the regulation of cellular homeostasis. Since the main sources of ROS and the related reactive nitrogen species (RNS) within living cells are mitochondria, dysfunctional mitochondria promote cell death by producing excessive fluxes of free radicals. Accumulating evidence shows that NRF2 signaling affects mitochondrial biogenesis *via* multiple mechanisms, such as controlling mitochondrial membrane potential and ATP synthesis, mitochondrial fatty acid oxidation, and the structural and functional integrity of the mitochondria (Dinkova-Kostova and Abramov, 2015).

Although direct evidence has not been demonstrated conclusively, several studies indicate that NRF2 activation contributes to the amelioration of mitochondrial dysfunction, particularly under conditions of oxidative stress, leading to suppression of accelerated cell apoptosis (Kang et al, 2020; Xiao et al, 2017; Yang et al, 2018).

In 1999, it was reported using a modified yeast two-hybrid system that NRF2 is a novel caspase substrate (Ohtsubo et al, 1999). Activation of caspases is the core machinery of apoptosis. Although the biological significance of this signaling crosstalk has not been followed by studies *in vivo*, this finding suggested possible interactions between NRF2 and critical factors regulating cell apoptosis early in the history of NRF2 research. Association between NRF2 and cyclin-dependent kinase inhibitor 1 (p21) pathways in cell apoptosis has been suggested in several studies. p21 is a cyclin-dependent kinase inhibitor that regulates many cellular processes, such as cell cycle, DNA replication and repair, cell differentiation, senescence, and apoptosis.

Direct molecular interaction between NRF2 and p21 was described (Chen et al, 2009), and several follow-up studies have been published (Han et al, 2019; Han et al, 2018; Li et al, 2018; Villeneuve et al, 2009). Limited *in vitro* evidence indicates that the downstream action of the NRF2-p21 axis affecting cell apoptosis likely occurs through regulation of redox homeostasis. Further studies using *in vivo* models are required to elucidate the physiological significance of this crosstalk.

Transformation related protein 53 (p53) is a transcription factor activated by DNA damage, leading to cell cycle arrest allowing for repair of DNA damage. When DNA damage remains unrepaired, p53 plays a critical role in the induction of apoptosis. Faraonio et al (2006) reported that p53 suppresses the NRF2-dependent transcription of ARE-containing promoters of antioxidant genes. Further, Chen et al (2012b) showed that the p53 signaling pathway can induce or inhibit the NRF2 pathway depending on intracellular ROS levels. Although both studies hypothesized that crosstalk between NRF2 and p53 may coordinate cell survival and p53-dependent apoptosis, direct evidence has not yet been demonstrated in non-cancer models.

Crosstalk between NRF2 and AhR has been studied, which is at least partially associated with cell death (Esakky et al, 2015). Joo et al (2013) demonstrated that miR-125b is transcriptionally regulated by NRF2, and it serves as an inhibitor of the AhR repressor (AhRR), which, in turn, regulates AhR and p53 signaling. In *Nrf2* knockout mice, an adaptive elevation of miR-125b by cisplatin treatment was suppressed, causing aggravated kidney toxicity and elevated tubular cell apoptosis.

In cancer cells, apoptotic cell death has a unique signature, and its association with NRF2 is also different from that in normal cells. The common hallmarks of cancer cells are uncontrolled growth, angiogenesis, and evasion of apoptosis. Thus, apoptosis-inducing therapies are one of the effective non-surgical cancer treatment strategies. In many types of cancer cells, NRF2 acquires a stable overexpression phenotype, leading to dysregulated cell proliferation and resistance to anti-cancer drugs (Homma et al, 2009), as well as an accelerated evasion to apoptosis (Jiang et al, 2020).

*NRF2* knockdown in non-small cell lung cancer cell lines promoted radiation-induced cell apoptosis, where interestingly, NOTCH1 expression under radiation exposure was decreased significantly by depletion of *NRF2*, implying that NOTCH-NRF2 signaling crosstalk might be correlated with apoptosis resistance in cancer cells (Zhao et al, 2016). p62 plays a key role in the regulation of cell proliferation and survival, and it has been identified as a tumor suppressor. Jiang et al (2020) reported that p62 promotes cell proliferation, apoptosis resistance, and invasion of prostate cancer cells along with elevated NRF2 activity in a prostate cancer cell line. Niture and Jaiswal (2012) demonstrated direct transcriptional regulation of *Bcl2* by NRF2 in mice and human hepatoma cell lines. Further, it was observed that NRF2-mediated upregulation of BCL2 suppressed chemotherapy- and radiation-induced DNA fragmentation and apoptosis.

In addition, some novel NRF2 inhibitors have been reported to induce apoptosis in cancer cells (Zhang et al, 2017). Since drug resistance is a major obstacle for chemotherapy, apoptosis targeted therapy—especially targeting NRF2 to inhibit its dysregulated activation—has attracted attention. However, the specificity of current NRF2 inhibitors is limited. Thus, there is a long way for a clinical application of NRF2 inhibitors to cancer treatment.

### Autophagic cell death

Autophagy is the process that delivers cytoplasmic material of endogenous or exogenous origin to the lysosome for degradation (Galluzzi et al, 2017). In general, autophagy is considered as a pro-survival process contributing to cellular homeostasis in response to several types of cellular stresses by eliminating aged proteins and damaged organelles. In addition to the typical understanding of autophagy as an adaptive process, increasing evidence links its association to cell death processes during development and pathogenesis in a manner distinct from apoptosis (Denton and Kumar, 2019).

Crosstalk between the NRF2 and autophagic pathways has been well recognized. p62 is an autophagy substrate, serving as cargo receptors involved in selective autophagy. p62 dampens NRF2 signaling activity *via* a direct, interfering interaction with KEAP1 (Komatsu et al, 2010; Lau et al, 2010). Nrf2 also promotes the ARE-dependent expression of p62 (Jain et al, 2010). The biological significance of the NRF2-autophagy axis has been studied widely, suggesting important roles, such as maintenance of the hepatic redox system (Taguchi et al, 2012) and the cytoprotective effects from the distinct types of damage in hepatocytes, cardiomyocytes, neural cells, and among other cell types (Lv et al, 2019; Wu et al, 2021; Zhang et al, 2021).

Interestingly, a recent study has shown that crosstalk between autophagy and NRF2 signaling is likely to be critical in mammalian survival. *Atg7*-deficient mice are born developmentally normal but fail to survive whereas co-deletion of *p53* and *Atg7* remarkably extended their life span. By contrast, *Nrf2* and *Atg7* double-knockout mice died rapidly due to small intestine damage, which was not rescued by *p53* co-deletion. These observations indicate the contribution of autophagy to mouse survival and its functional inter-dependencies on p53 and NRF2 (Yang et al, 2020).

Cigarette smoke has been reported to induce autophagy in airway epithelial cells. Autophagic cell death seems to be a central pathogenic process in chronic obstructive pulmonary disease. Cigarette smoke extract induces expression of both LC3B-I and LC3B-II as well as autophagosomes in airway epithelial cell lines, which was suppressed by genetic activation of NRF2 (Zhu et al, 2013). Elevated cellular GSH levels likely underlie NRF2-mediated inhibition of accelerated autophagy by cigarette smoke.

The therapeutic effects of urolithin B (UB), a gut microbiota metabolite, were studied using *in vivo* and *in vitro* myocardial ischemia/reperfusion models. It was demonstrated that UB ameliorated cardiac damage by inhibiting autophagy, leading to suppression of caspase3-dependent cell apoptosis. The protective effects by UB also involved NRF2 activation, which was likely through the p62-KEAP1 axis (Zheng et al, 2020).

In cancer cells, modulation of autophagy plays dual roles, that is, suppression and promotion of cancers depending on cellular context. Specifically, a pro-death function of autophagy in the setting of cancer cell treatment has been attracting attention as an alternative treatment strategy for apoptosis-resistant cancer cells. Temozolomide is a cytotoxic drug that induces significant autophagic cell death in glioblastoma multiforme cells. *NRF2*-knockdown enhanced the basal level of autophagy in this cell line. Further, temozolomide treatment of *NRF2*-knockdown cells showed lower viability along with elevated autophagy (Zhou et al, 2013). *NRF2* suppression may increase sensitivity of glioma cells to temozolomide *via* autophagy. Park et al (2012) demonstrated that human lung carcinoma A549 cells treated with selenium displayed not only caspase-dependent apoptosis, but also an elevation in autophagic flux as well as NRF2 activation contributing to cancer cell survival.

Although the association between autophagy, NRF2 signaling, and cancer cell fate was not investigated in this study, accelerated autophagic flux in cancer cells harboring constitutive NRF2 activation (mediated by a somatic mutation of the *KEAP1* gene and/or epigenetic alteration of *KEAP1* promoter) may underlie some chemotherapeutic resistance. It is also interesting to note that tumor resistance to radiotherapy is often associated with upregulation of autophagy in many types of cancer cells (Hu et al, 2016). Chen et al (2017b) demonstrated that radiation facilitates autophagic flux in a human osteosarcoma cell line, leading to NRF2 activation, which, in turn, provides a protective role against apoptosis in irradiated cells. These findings highlight the complex role of NRF2 and autophagy working in concert in cancer cells, to facilitate suppression or enhancement of cell death.

### Ferroptosis

Ferroptosis is a type of cell death recognized in the early 2000s (Jiang et al, 2021), which is distinct from apoptosis and occurs in the absence of any known genetically-encoded death mechanism. Ferroptosis is an oxidative-stress-induced form of cell death associated with two biochemical features, iron accumulation and lipid peroxidation.

Recent studies have implicated the tight association between ferroptosis and pathophysiological processes of many diseases, such as organ injuries and degenerative diseases (*i.e*., ischemic organ damage, liver and lung fibrosis, neurodegeneration) and several types of cancers. Therefore, regulation of ferroptosis has gained attention with respect to clinical applications, whereas the physiological role of ferroptosis is still largely unknown.

Since NRF2 target genes include several genes related to ferroptosis, such as genes related to iron regulation (heme synthesis, hemoglobin catabolism, iron storage, and iron export), as well as genes for GSH regulation and NADPH regeneration (Kerins and Ooi, 2018), the association between NRF2 pathway and ferroptosis has been studied (Dodson et al, 2019). Ferroptosis drives cardiomyopathy induced by chemotherapy (doxorubicin) in the mouse, where upregulation of *Hmox1* seems important (Fang et al, 2019); this ferroptotic cardiomyopathy was abolished in *Nrf2*-deficent mice.

Using a mouse model of acute lung injury induced by intestinal ischemia/reperfusion, Dong et al (2020) demonstrated that the pathophysiology of acute lung injury was at least partially driven by ferroptosis. After intestinal ischemia/reperfusion, ferroptosis-mediated cellular morphological hallmarks were observed in the lung tissues of wild-type mice that were more severe in *Nrf2* knockout mice. However, the acute lung injury in the *Nrf2* knockout mice was reduced by administration of a ferroptosis-specific inhibitor, highlighting the association between NRF2 and ferroptosis-mediated lung damage. A link between NRF2 and ferroptosis in neurodegenerative diseases has been hypothesized but not well established. Recently, La Rosa et al, (2021) reported that in fibroblasts obtained from skin biopsies and leukocytes isolated from blood of patients with the neurodegenerative disease, Friedreich's Ataxia showed suppressed expression of NRF2 and its activity. Friedreich's Ataxia is caused by the decreased expression of the mitochondrial protein frataxin, which, in turn, is associated with ferroptosis. In normal cells, NRF2 seems to upregulate anti-ferroptotic defenses, which may induce protective effects against ferroptosis-mediated diseases (Abdalkader et al, 2018).

Multiple studies have revealed that numerous cancer-relevant genes and signaling pathways regulate ferroptosis. In this setting, the association of ferroptosis with NRF2 signaling has been studied. Artesunate, an anti-malaria drug, exhibits anti-tumor activity by inducing cell death *via* ROS-mediated ferroptosis. Using a head and neck cancer cell line, the role of NRF2 signaling and resistance of cells to artesunate was examined (Roh et al, 2017). Artesunate activates NRF2 activity leading to ferroptosis resistance that can be reversed by *NRF2* silencing. Sun et al (2016) used a hepatocellular carcinoma cell line to show that treatment with ferroptosis-inducing compounds (*e.g.*, erastin, sorafenib, and buthionine sulfoximine) upregulated NRF2 target genes *via* p62- and KEAP1-dependent regulatory pathways, contributing to ferroptosis resistance. In cancer cell lines, p53 sensitizes cells to ferroptosis by repressing transcription of the *xCT* or *SLC7A11* gene (Jiang et al, 2015a).

Chen et al (2017a) demonstrated that ARF, a tumor suppressor protein, directly interacts with NRF2 both *in vitro* and in the xenograft tumor model, where loss of ARE induces NRF2 activation followed by elevation of *SLC7A11* expression and promotion of resistance to ROS-induced ferroptosis and cancer cell survival in p53 null cells. Hassannia et al (2018) reported withaferin A as a natural ferroptosis-inducing agent in neuroblastoma, whose mechanism includes the canonical ferroptosis induction pathway (inactivation of glutathione peroxidase 4 [GPX4]) as well as the non-canonical pathway involving NRF2 signaling.

Withaferin A activates NRF2 through a KEAP1 independent mechanism, resulting in increased intracellular labile Fe(II) on excessive activation of HMOX1, which, in turn, is sufficient to induce lipid peroxidation and ferroptosis. Further, a recent study using three-dimensional spheroids of a lung cancer cell line demonstrates that NRF2 overexpression is likely to be necessary for the survival of cancer spheroids *via* suppression of ferroptosis (Takahashi et al, 2020). In this study, Takahashi et al observed that downregulation of NRF2 accelerated lipid peroxidation followed by ferroptosis and cell death in the inner cells of the spheroid structure. Interestingly, *NRF2* downregulation increased the expression of selenoproteins, including GPX4. However, in the unique cellular environment observed in *NRF2* knockdown spheroids, such as high levels of ROS and low GSH content, upregulated GPX4 expression seems not to contribute to alteration of the high vulnerability to ferroptosis.

In summary, accumulating evidence strongly indicates that NRF2 signaling has multifaceted integration into the process of ferroptosis in cancer cells depending on the microenvironment and types and states of cancer cells. Thus, careful consideration of study models, active employment of *in vivo* models, and a multi-layered understanding of molecular mechanisms must be undertaken in future studies.

## Conclusions

**FIG. 8.**
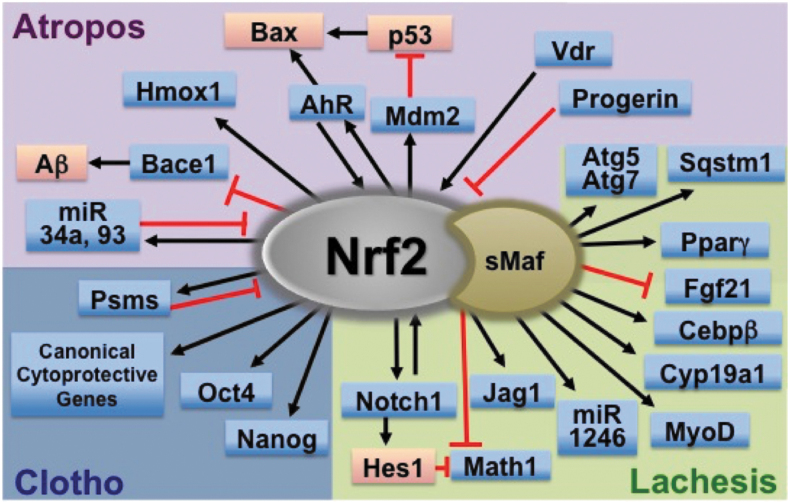
**NRF2 is integrated into signaling networks that regulate cell fates and functions.** Through direct- and indirect-signaling, NRF2 forms an expanded network encompassing a wide range of pathways to affect cell fate determination throughout the life cycle of cells. The major pathways affected by NRF2 within the three stages of the cell life cycle: cell birth (*Clotho*), cell differentiation (*Lachesis*), and cell death (*Atropos*) are summarized. Note that the overarching biological functions of these signaling pathways and their downstream factors can contribute to multiple aspects of cell fate outcomes, and they are not fully addressed in this summary figure. The *blue* and *orange boxes* indicate the molecules playing roles as first and second messengers in a pathway, respectively. The *black arrows* indicate positive regulation, and the *red lines* negative regulation. Aβ, amyloid β; Atg5, 7, autophagy related 5, 7; Bace1, B-site amyloid precursor protein cleaving enzyme 1; Bax, Bcl-2 associated X-protein; Cebpβ, CCAAT/enhancer-binding protein-β; Cyp19a1, cytochrome P450 family 19 subfamily a member 1; Fgf21, fibroblast growth factor 21; Hes1, hairy and enhancer of split 1; Hmox 1, heme oxygenase 1; Jag1, jagged canonical Notch ligand 1; Math1, (Atoh1) atonal bHLH transcription factor 1; Mdm2, murine double minute 2; MyoD, myogenic differentiation 1; Oct4, POU domain class 5 transcription factor 1; Pparγ, peroxisome proliferator-activated receptor γ; Psms, proteasome subunits; Sqstm1, sequestosome 1.

- NRF2 signaling interfaces with the processes of cellular transitions and determinations in the three major phases of the life cycle of the cell.
- *CLOTHO*: NRF2 influences maintenance, proliferation (self-renewal), and early transitions of stem cells (and stem cell niches).
- *LACHESIS*: NRF2 fine-tunes cell differentiation and proliferation in multiple types of cells and tissues.
- *ATROPOS*: NRF2 suppresses and/or accelerates cell senescence and programmed cell death processes and protects against cytotoxic insults.
- Molecular integration of NRF2 into cell fate regulatory mechanisms occurs *via* the canonical NRF2-target genes regulating cellular redox status, which, in turn, affects multiple layers of cell fate determination, and more newly recognized NRF2 target genes that directly or indirectly affect cell fate outcomes.
- Overlapping responsiveness of ARE(s) to various transcription factors beyond NRF2 results in a multiplicity of responses in ARE-driven gene expression. This regulatory compensation of ARE-driven genes under loss of Nrf2-function implies a supportive role of NRF2 in cell fate determinations.
- NRF2 signaling engages in extensive crosstalk across a wide range of transcriptional cascades and signaling pathways (Fig. 8), providing a multifactorial signaling network to modify cell fate regulatory processes.
- Since cell fate commitment is a dynamic and intricate series of biological process governed by multiple components, a singular focus on NRF2 signaling limits better understandings of the complex underlying molecular mechanisms. The near full lifespan of *Nrf2* knockout mice (in the absence of stressors) likely precludes a central role of NRF2, but an extensive literature using *in vitro* systems supports a facilitative and modulatory one. Future studies are warranted with the use of *in vivo* models optimized to approach the roles of NRF2 signaling within the complexity of compensatory and crosstalk signaling in cell fate determinations.

## Abbreviations Used

Aβ: amyloid β
AhR: aryl hydrocarbon receptor
ARE: antioxidant response element
ARNT: aryl hydrocarbon receptor nuclear translocator
ATF: activating transcription factors
ATG5: 7 Autophagy related 5, 7
BACE1: B-site amyloid precursor protein cleaving enzyme 1
BACH: BTB and CNC homology basic leucine zipper transcription factor
BAX: Bcl-2 associated X-protein
BMP: bone morphogenetic protein
CEBPβ: CCAAT/enhancer-binding protein-β
ChIP: chromatin immunoprecipitation
*c-Jun/Jun*: Jun proto-oncogene
CNC: cap'n'collar
CNTF: ciliary neurotrophic factor
CRE: cAMP (cyclic adenosine monophosphate) response elements
CREB: cAMP-responsive element binding protein
CREM: cAMP response element modulator
CYP19A1: cytochrome P450 family 19 subfamily a member 1
ED: embryonic day
EGFR: epidermal growth factor receptor
EPC: endothelial progenitor cell
ESC: embryonic stem cell
FGF21: fibroblast growth factor 21
*Fos*: Fos proto-oncogene
FRA: Fos-related antigen
G6PD: glucose-6-phosphate dehydrogenase
GCL: glutamate cysteine ligase
GCLC: glutamate-cysteine ligase, catalytic subunit
GPX4: glutathione peroxidase 4
GSH: glutathione
GSS: glutathione synthetase
GSS: glutathione synthase
HDAC4: histone deacetylase inhibitor 4
HES1: hairy and enhancer of split 1
hESC: human embryonic stem cell
HGPS: Hutchinson-Gilford progeria syndrome
HIF: hypoxia-inducible factor
HMOX1: heme oxygenase 1
HRE: hypoxia response element
HSPC: hematopoietic stem/progenitor cell
ID: inhibitor of DNA-binding proteins
IDH1: isocitrate dehydrogenase 1
iPSC: induced pluripotent stem cell
JAG1: Jagged canonical Notch ligand 1
JAK: Janus kinase
KEAP1: kelch-like ECH-associated protein 1
LIF: leukemia inhibitory factor
LIFR: LIF-receptor
MARE: Maf homodimer specific binding sites
MATH1: (Atoh1) atonal bHLH transcription factor 1
MDM2: murine double minute 2
ME1: malic enzyme 1
MEF: mouse embryo fibroblast
miRNA: microRNA
MSC: multipotent stem cell
MTHFD2: methylenetetrahydrofolate dehydrogenase 2
MYOD: myogenic differentiation 1
MYOG: myogenin
NANOG: Nanog homeobox protein
NPC: neural stem/progenitor cell
NQO1: NAD(P)H dehydrogenase, quinone 1
NRF2: NF-E2-related factor 2
NRL: neural retina-specific leucine zipper protein
OCT4: POU class 5 homeobox 1
P: postnatal day
p21: cyclin-dependent kinase inhibitor 1
p53: transformation related protein 53
p62: sequestosome 1
PGD: 6-phosphogluconate dehydrogenase
PHGDH: phosphoglycerate dehydrogenase
PPAR: peroxisome proliferator-activated receptor
PPARγ: peroxisome proliferator-activated receptor γ
PPAT: phosphoribosyl pyrophosphate amidotransferase
PSAT1: phosphoserine aminotransferase 1
PSMS: proteasome subunits
PTEN: phosphatase and tensin homolog deleted from chromosome 10
RAR: retinoic acid receptor
RARE: retinoic acid response element
ROS: reactive oxygen species
RXR: retinoid X receptor
SHMT2: serine hydroxymethyltransferase 2
SIRT1: sirtuin1
SMAD: suppressor of mothers against decapentaplegic
sMAF: small Maf
SP-A: surfactant protein A
SQSTM1: sequestosome 1
STAT3: signal transducer and activator of transcription 3
TA: transit-amplifying progenitor cells
TALDO1: transaldolase 1
Thyroid RE: thyroid hormone response element
TKT: transketolase
TR: thyroid hormone receptor
TRE: TPA (12-O-tetradecanoylphorbol-13-Acetate)-responsive element
TRX2: thioredoxin-2
TRXR1: thioredoxin reductase 1
TRXR1: thioredoxin reductase-1
TXN: thioredoxin
UB: urolithin B
VDR: vitamin D_3_ receptor
VDRE: vitamin D response element
xCT: cystine/glutamate antiporter (SLC7A11)
